# Trends in Correlation-Based Pattern Recognition and Tracking in Forward-Looking Infrared Imagery

**DOI:** 10.3390/s140813437

**Published:** 2014-07-24

**Authors:** Mohammad S. Alam, Sharif M. A. Bhuiyan

**Affiliations:** 1 Department of Electrical and Computer Engineering, University of South, Alabama Mobile, AL 36688-0002, USA; 2 Department of Electrical Engineering, Tuskegee University, Tuskegee, AL 36088, USA; E-Mail: bhuiyans@mytu.tuskegee.edu

**Keywords:** patter recognition, target tracking, forward looking infra-red (flir) imagery, correlation filter

## Abstract

In this paper, we review the recent trends and advancements on correlation-based pattern recognition and tracking in forward-looking infrared (FLIR) imagery. In particular, we discuss matched filter-based correlation techniques for target detection and tracking which are widely used for various real time applications. We analyze and present test results involving recently reported matched filters such as the maximum average correlation height (MACH) filter and its variants, and distance classifier correlation filter (DCCF) and its variants. Test results are presented for both single/multiple target detection and tracking using various real-life FLIR image sequences.

## 1. Introduction

Pattern recognition deals with the detection and identification of a desired pattern or target in an unknown input scene, which may or may not contain the target, and the determination of the spatial location of any target present. In pattern recognition or classification, the input is an image while the output is a decision signal based on some characteristic features of the input image. The number of features is usually fewer than the total necessary to describe the complete target of interest, and this leads to a loss of information. Because of the crucial role of decision making required in pattern recognition, it is fundamentally an information reduction process, whereby, it is not possible to reconstruct the pattern but it is possible to give a precise decision [1–3].

Although a great deal of effort has been expended on detecting objects in visual images, only limited amount of work has been reported on the detection and tracking of targets in infrared images. In general, existing methods on infrared images work for a limited number of situations due to various practical constraints. An infrared sensor detects infrared radiation and converts it to an image by converting the temperature difference between an object and the surrounding background. The temperature scale is converted into a color scale or a gray scale on a display and in this way an image is obtained. This type of sensor or camera can image an object through smoke in a burning house, heat leaking from a house or objects in the absence of any reflected light (at night) [4]. The images captured by infrared sensors are becoming an integral part of the ongoing research on automatic target recognition (ATR).

Forward-looking infrared (FLIR) images are frequently used in ATR applications. The detection and discrimination of targets in infrared imagery has been a challenging problem due to low signal-to-noise ratio (SNR) and the variability of target and clutter signatures. The FLIR sequences, tested in this work, are recorded from a moving platform and include independently moving objects under various distortions and background variations. Thus, sensor ego-motion and object motions induce coupled motions into the FLIR images, which make the detection and tracking of the objects extremely complicated. To detect independently moving objects in FLIR image sequences, the sensor properties must also be taken into account.

Real life FLIR imagery demonstrates a number of well-known challenges such as significantly high level of variability of target thermal signatures, size/aspect, locations within the scene; large number of target classes; lack of prior information; obscured targets; competing cluttered background scenery; different geographic, meteorological and weather conditions; time of the day; high ego-motion; sensor noise; and variations caused by translation, rotation, and scaling of the targets. Furthermore, inconsistencies in the signature of targets, similarities between the signatures of different targets, limited training and testing data, camouflaged targets, non-repeatability of target signatures, and difficulty in exploiting contextual information make the recognition problem even more challenging in target detection in FLIR imagery. In the case of FLIR images, additional challenges are caused due to the following important differences [5–7] with visual sequences:

- The thermal images are obtained by sensing the radiation in the infrared spectrum, which is either emitted or reflected by the object in the scene. Due to this property the images obtained from an infrared sensor have extremely low SNR, which results in limited information for performing detection or tracking task.
- FLIR imagery smoothes out object edges and corners leading to a reduction of distinct features.
- The generation and maintenance of kinetic energy usually heats up a moving object (e.g., friction, engine combustion). Consequently, moving objects often appear brighter than the background.
- FLIR images are noisy and have less contrast. Moreover, they often contain dirt on the lens, or local sensor failure at certain pixel locations.
- FLIR sequences are not easily available (especially not from controlled experiments) and have a lower resolution. The sequences available to us are 128 × 128 pixels as compared to the 512 × 512 pixels, and more, of standard visual cameras.
- FLIR sequences are often under difficult circumstances and may have abrupt discontinuities in motion.

Due to the limitations and difficulties of FLIR imagery, they demand more robust techniques than visual sequences. This paper presents some widely used pattern recognition and target tracking techniques adopted for FLIR imagery. In this paper, we discuss several target detection and tracking algorithms which are based on the recently reported matched filter-based correlation techniques such as the MACH, EMACH, DCCF, and PDCCF filters. The performance of these algorithms was tested using real life FLIR image sequences supplied by the Army Missile Command.

## 2. Matched Filter-Based Correlation

The matched filter-based correlator was first introduced in 1964 [8]. In this technique, the input signal *f*(σ,*ε*) is Fourier transformed to yield

(1) F(ο,υ)=ℑ[f(σ,ɛ)]

where ℑ represents the Fourier transform operation, σ and *ε* represents the spatial domain variables, and σ and υ represents the frequency domain variables, respectively. The correlation output is obtained by inverse Fourier transform operation given by

(2) $$g(\sigma ,ɛ)={ℑ}^{-1}[F(ο,\upsilon )H(ο,\upsilon )]$$

where, ℑ ^−1^ represents 2D inverse Fourier transform operation.

If the input object is moved laterally in the input plane, the Fourier transform remains fixed in space but is multiplied by a phase factor that depends on the lateral movement. Therefore, the coordinates of the bright correlation output is proportional to the coordinates of the signal *f*(σ,*ε*) located at the input plane. The intensity of the bright correlation spot is proportional to the degree to which the input and the filter functions are matched. This is also valid for multiple objects present at different locations of the input plane. This correlation system provides a great deal of sensitivity since it is both phase matched and amplitude matched [9–11].

For the complex matched filter (CMF), the frequency plane filter function is expressed by

(3) $$H_{\text{cmf}}=R^{*}(ο,\upsilon )=|R(ο,\upsilon )|\exp[-j\phi (ο,\upsilon )]$$

where, |*R*(*ο*,*υ*)| is the amplitude and φ(*ο*,*υ*) is the phase factor of the Fourier spectrum of the reference function *r*(σ,*ε*). When the input is similar to *r*(σ,*ε*), the phase variation is canceled at the Fourier plane, thus producing a plane wave of light. The correlation peak corresponding to CMF is not very sharp due to the squaring of the magnitude of *r*(σ,*ε*). Consequently, the resulting diffraction efficiency of a CMF is very poor. This filter is also unacceptably sensitive to even small changes in the reference signal or image. Currently available spatial light modulators (SLMs) cannot accommodate the full complex frequency response needed by CMFs.

## 3. Phase Only Filter (POF)

The optimum case with respect to light efficiency for a matched filter is realized by a phase only filter (POF) structure [10]. This filter is obtained by omitting the amplitude information and is defined as

(4) $$H_{\text{pof}}(ο,\upsilon )=\frac{R^{*}(ο,\upsilon )}{\left|R(ο,\upsilon )\right|}=\exp[-j\phi (ο,\upsilon )]$$

Besides the improvement in light efficiency, the correlation peak intensity is also enhanced with a POF. However, although the autocorrelation peak intensity is higher than that of a CMF, it is not as sharp as might be produced by an IF since the product *H*_pof_(*ο*,*υ*)*R*(*ο*,*υ*) is not necessarily a constant.

## 4. Amplitude-Modulated Phase Only Filter (AMPOF)

Amplitude modulated phase only filter (AMPOF) [12] is given by

(5) $$H_{\text{ampof}}(ο,\upsilon )=\frac{A}{B+\left|R(ο,\upsilon )\right|}\exp[-j\phi (ο,\upsilon )]$$

where *A* and *B* are either constants or functions of *ο* and *υ*. The gain control factor *A* guarantees that the transmittance of the filter is less than unity. With the inclusion of *B*; the pole problem is solved and at the same time it is possible to yield very high autocorrelation peak.

## 5. Synthetic Discriminant Functions (SDF)

The SDF-based correlation filters had shown robust performance for distortion tolerant pattern recognition [13–15]. Assume *x*_1_(σ,*ε*), *x*_2_(σ,*ε*), …, *x_N_*(σ,*ε*) denote *N* training images representing possible distortions to a reference image *x*(σ,*ε*). The 2D Fourier transform of *x*(σ,*ε*) may be expressed as

(6) X(ο,υ)=∫∫x(σ,ɛ)exp[−j2π(οσ+υɛ)]×dσdɛ

A composite image *h*(σ,*ε*) is designed from the training images such that when the complex conjugate of its Fourier transform, denoted as H*(*ο*,*υ*), is correlated with input Fourier transform, a similar output is obtained for all *N* inputs, *x*_1_(σ,*ε*), *x*_2_(σ,*ε*), …, *x_N_*(σ,*ε*). This type of correlator is known as frequency plane correlator. For this filter, the resulting spatial domain correlation output *c*(*τ_σ_*,*τ_ε_*) may be expressed as [13]

(7) $$\begin{array}{ll} c(\tau_{\sigma },\tau_{ɛ}) & =\int \int F(ο,\upsilon )H^{*}(ο,\upsilon )\exp[j2\pi (ο\tau_{\sigma }+\upsilon \tau_{ɛ})]\text{d}ο\text{d}\upsilon \\ & =\int \int h^{*}(\sigma ,ɛ)f(\sigma +\tau_{\sigma },ɛ+\tau_{ɛ})\text{d}\sigma \text{d}ɛ\\ & =h(\sigma ,ɛ)⊙f(\sigma ,ɛ) \end{array}$$

where ⊙ denotes a two-dimensional cross-correlation operation.

In the Equal Correlation Peak SDF (ECP-SDF) design, the objective is to select a filter impulse response *h*(σ,*ε*) such that the resulting crosscorrelations with all the *N* training images are the same, which is impossible in practice. Hester and Casasent [13] introduced a technique that requires that only the values at the origin of these crosscorrelations should be the same as shown in the following equation,

(8) $$\begin{array}{cc} {h(\sigma ,ɛ)⊙x_{i}(\sigma ,ɛ)|}_{\tau_{\sigma }=0,\tau_{ɛ}=0} & =\int \int h^{*}(\sigma ,ɛ)x_{i}(\sigma ,ɛ)\text{d}\sigma \text{d}ɛ\\ & \begin{array}{cc} =c, & i=1,2,\ldots \ldots .N \end{array} \end{array}$$

where *c* is a prespecified constant. Equation (8) shows that a *h*(σ,*ε*) would yield the same constant value *c* at the origin (location of the autocorrelation peak) for all *N* training images (*i.e.*, *x*_1_(σ,*ε*) to *x_N_*(σ,*ε*)). When the input is a non-training image from the same class, the crosscorrelation output at the origin will be similar to this constant *c* and it can be recognized. The success of this approach depends on selecting a proper training set.

Assume that *h*(σ,*ε*) is a linear combination of the *N* training images, given by

(9) $$h(\sigma ,ɛ)=a_{1}x_{1}(\sigma ,ɛ)+\ldots \ldots \ldots \ldots ..a_{N}x_{N}(\sigma ,ɛ)$$

where the coefficients *a*_1_, *a*_2_, … , *a_N_* are determined in a way to satisfy the constraints of Equation (8). Substituting Equation (9) into Equation (8), we get

(10) $$\sum_{i=1}^{N}a_{i}^{*}R_{ij}=c,j=1,2,\ldots \ldots ,N,$$

where

(11) $$R_{ij}=\iint x_{i}^{*}(\sigma ,ɛ)x_{j}(\sigma ,ɛ)\text{d}\sigma \text{d}ɛ$$

is the inner product, *i.e.*, the crosscorrelation at the origin of the training images *x_i_*(σ,*ε*) and *x_j_*(σ,*ε*). If the training images are real, then there is no need for the conjugate operation shown in Equation (11). Equation (10) represents *N* complex linear equations with *N* complex unknowns, *a*_1_, *a*_2_, …, *a_N_*, respectively. These equations can be solved by using any standard techniques, such as Gauss-Seidel Elimination method.

## 6. Modified Synthetic Discriminant Functions

The ECP-SDF is designed to produce a value *c_i_* at the origin of the output plane when the *i*-th training image is used as the input. However, there are some practical problems in using this filter for pattern recognition applications [16–18]. Consequently, some improvements on ECP_SDF are suggested which are introduced in the following subsections.

### 6.1. Generalized SDF

Assume that the training images *x*_1_(σ,*ε*), *x*_2_(σ,*ε*), …, *x_N_*(σ,*ε*) are sampled to yield arrays *x*_1_(*m*,*n*), *x*_2_(*m*,*n*), …, *x_N_*(*m*,*n*) each with ρ *=* ρ_1_ρ_2_ pixels, where ρ_1_ is the number of pixels in the vertical direction while ρ_2_ is the number of pixels in the horizontal direction of each image. It is also assumed that ρ-dimensional column vectors **x**_1_, **x**_2_, …, **x***_N_* are obtained by placing the elements in these training images in vectors where the scanning direction is from left to right and from top to bottom. Similarly, the ρ-dimensional column vector **h** is used to denote the composite image *h*(*m*,*n*). Then the constraints in Equation (8) can be rewritten as

(12) $$\begin{array}{cc} {\text{h}}^{+}{\text{x}}_{i}=c_{i}, & i=1,2,3,\ldots \ldots .N \end{array}$$

where the superscript ^+^ denotes the conjugate transpose operation. The data matrix **X** is assumed to have the vector **x***_i_* as its *i*-th column and is thus a ρ × *N* matrix. It is also assumed that ρ ≫ *N*, *i.e.*, the number of pixels in the training images is much larger than the number of training images, and that the columns of this matrix are linearly independent. Using this notation, Equation (12) can be rewritten as

(13) $${\text{X}}^{+}\text{h}={\text{c}}^{*}$$

The ECP-SDF assumes that the composite image **h** is of the following form

(14) h=Xa

where **a** is the vector of coefficients. Substituting Equation (14) into Equation (13) and solving for **a** yields

(15) $$\text{a}={\left({\text{X}}^{+}\text{X}\right)}^{-1}{\text{c}}^{*}$$

The filter vector **h** can be obtained by substituting Equation (15) into Equation (14) to get

(16) $${\text{h}}_{\text{ECP}}=\text{X}{\left({\text{X}}^{+}\text{X}\right)}^{-1}{\text{c}}^{*}$$

A general expression for **h** satisfying Equation (13) is given by

(17) $${\text{h}}_{\text{GSDF}}=\text{X}{\left({\text{X}}^{+}\text{X}\right)}^{-1}{\text{c}}^{*}+[{\text{I}}_{d}-\text{X}{\left({\text{X}}^{+}\text{X}\right)}^{-1}{\text{X}}^{+}]\text{z}$$

where **I_d_** is the ρ × ρ diagonal identity matrix and **z** is any column vector with ρ complex entries. The ECP-SDF is obtained from Equation (17) when **z** = 0. The filter vector in Equation (17) is known as the generalized SDF [17].

### 6.2. Minimum Variance SDF

Consider a situation where the input image is one of the training images **x***_i_* **c**orrupted by additive noise **n**. Then the resulting output value *y*, *i.e.*, the value of the crosscorrelation at the origin is given by

(18) $$\begin{array}{c} y={\text{h}}^{+}({\text{x}}_{i}+\text{n})\\ =c_{i}+{\text{h}}^{+}\text{n} \end{array}$$

where **h** is designed to satisfy Equation (12). From Equation (18) it is evident that the output *y* is the desired output *c_i_* corrupted by the random variable **(h^+^n)**. The minimum variance synthetic discriminant function (MVSDF) [18] attempts to design **h** such that the variance in the output caused by input noise is minimized while satisfying the constraints in Equation (13).

Assume that the real noise vector **n** is a zero-mean vector with a ρ *×* ρ covariance matrix Σ. The variance of *y* corresponding to **h^+^n** can be expressed as

(19) $$\sigma_{y}^{2}=E\left\{{\left|{\text{h}}^{+}\text{n}\right|}^{2}\right\}=E\left\{{\text{h}}^{+}\text{n}{\text{n}}^{+}\text{h}\right\}={\text{h}}^{+}\sum \text{h}$$

It is desired that $\sigma_{y}^{2}$ in Equation (19) is as small as possible, which will ensure that the output values are close to the constrained values even in the presence of noise. Minimizing $\sigma_{y}^{2}$ in Equation (19) subject to the constraints in Equation (13) leads to the following MVSDF [18]

(20) $${\text{h}}_{\text{MVSDF}}=\sum^{-1}\text{X}{\left({\text{X}}^{+}\sum^{-1}\text{X}\right)}^{-1}{\text{c}}^{*}$$

This MVSDF is indeed optimal from noise tolerance considerations. One difficulty in using this MVSDF is that often Σ is not known. Even when it is known, it is impossible to calculate its inversion. Another problem is that the MVSDF controls only one point (the origin) in the output-correlation plane. Thus, large sidelobes may be observed in the correlation output.

### 6.3. Frequency-Domain SDFs

It is often more convenient to design the filters in the frequency domain [16–18]. Assume *f*(σ,*ε*) is the image and H*(*ο*,*υ*) is the complex filter function. Then the resulting correlation output *c*(*τ_σ_*,*τ_ε_*) at the origin is given by

(21) $$\begin{array}{cc} c(0,0) & =\iint H^{*}(ο,\upsilon )F(ο,\upsilon )\text{d}ο\text{d}\upsilon \\ & ={\hat{\text{h}}}^{*}\hat{\text{f}} \end{array}$$

where ˆ indicates that the corresponding vector or matrix is obtained by sampling frequency domain functions and the superscript ^+^ indicates a conjugate transpose operation. Because *c_i_*(0,0) is constrained to be *c_i_*, *i* = 1, 2, …, *N*, the constraints can be rewritten as

(22) $${\hat{\text{F}}}^{+}\hat{\text{h}}={\text{c}}^{*}$$

where $\hat{\text{F}}$ is a matrix with *N* columns with the *i*-th column containing ${\hat{\text{f}}}_{i}$. It is obvious that the Equations (13) and (22) are similar.

### 6.4. Minimum Average Correlation Energy (MACE) Filter

The correlation filters discussed so far control only one point in the correlation plane. For good location accuracy and discrimination, it is necessary to design filters capable of producing sharp correlation peaks. One such filter is the minimum average correlation energy (MACE) filter [19]. Assume *x*_1_(σ,*ε*), *x*_2_(σ,*ε*), …, *x_N_*(σ,*ε*) denote the *N* training images and *X*_1_(*ο*,*υ*), …, *X_N_*(*ο*,*υ*) denote their 2D Fourier transforms, respectively. If H*(*ο*,*υ*) denotes the transmittance of the filter function, then the filter may be constructed to satisfy the following condition.

(23) $$\begin{array}{cc} \iint X_{i}(ο,\upsilon )H^{*}(ο,\upsilon )\text{d}ο\text{d}\upsilon =c_{i}, & i=1,2,\ldots \ldots ,N \end{array}$$

In addition, the MACE filter minimizes the average correlation plane energy as shown below.

(24) $$\begin{array}{cc} E_{\text{ave}} & =\frac{1}{N}\sum_{i=1}^{N}\iint {\left|c_{i}(\tau_{\sigma },\tau_{ɛ})\right|}^{2}\text{d}\tau_{\sigma }\text{d}\tau_{ɛ}\\ & =\frac{1}{N}\sum_{i=1}^{N}\iint {\left|X_{i}(ο,\upsilon )\right|}^{2}{\left|H(ο,\upsilon )\right|}^{2}\text{d}ο\text{d}\upsilon \end{array}$$

By minimizing *E*_ave_, it is possible to keep the sidelobes in the correlation plane as low as possible. This is essentially an indirect attempt at reducing the problem of sidelobes. To carry out the minimization of *E*_ave_, the usual vector notation is used. If ${\hat{\text{x}}}_{i}$ denote the ρ-dimensional complex column vector obtained by sampling *X_i_*(*ο*,*υ*), then the constraints in Equation (23) can be rewritten as

(25) $${\hat{\text{X}}}^{+}\hat{\text{h}}={\text{c}}^{*}$$

where $\hat{\text{X}}$ is a ρ × *N* matrix with ${\hat{\text{x}}}_{i}$ as its *i*-th column. The *E*_ave_ in Equation (24) can be expressed as

(26) $$E_{\text{ave}}={\hat{\text{h}}}^{+}\hat{\text{D}}\hat{\text{h}}$$

where $\hat{\text{D}}$ is a ρ × ρ diagonal matrix. The entries along the diagonal are obtained by averaging |*X_i_*(*ο*,*υ*)|^2^, *i* = 1, 2, …, *N*, and then scanning the average from left to right and from top to bottom. Minimizing *E*_ave_ in Equation (26) subject to the constraints in Equation (25) leads to the following filter

(27) $${\hat{\text{h}}}_{\text{MACE}}={\hat{\text{D}}}^{-1}\hat{\text{X}}{\left({\hat{\text{X}}}^{+}{\hat{\text{D}}}^{-1}\hat{\text{X}}\right)}^{-1}{\text{c}}^{*}$$

In many simulation studies, filters designed using this approach produced sharp correlation peaks. However, MACE filters appear to have two drawbacks. The first is that there is no noise tolerance built into these filters. The second is that these filters seem to be more sensitive to intra-class variations. Casasent *et al.* [20] proposed Gaussian MACE filters to reduce the sensitivity of the MACE filters to intra-class variations. The idea behind Gaussian MACE filters is to reduce the sharpness of the resulting correlation peak and thus improve its noise tolerance. MACE filters appear to be the first set of composite filters that attempt to control the entire correlation plane.

### 6.5. Minimum Squared Error SDF (MSE-SDF) Filter

This SDF design approach yields better approximation of arbitrary output correlation shapes in the minimum squared error (MSE) sense over the MACE filter and this filter is termed as MSE-SDF [21]. Like MACE, this filter must satisfy the usual SDF constraint of Equation (25). Besides, in MSE-SDF, the filter function *H*(*ο*,*υ*) must make the correlation function *c_i_* approximate a prespecified desired shape *t_i_*, *i* = 1, 2, …, *N*. One measure of how well *c_i_* approximates *t_i_* is the average squared error *E* defined as

(28) $$\begin{array}{cc} E & =\frac{1}{N}\sum_{i=1}^{N}\iint {\left|t_{i}(\tau_{\sigma },\tau_{ɛ})-c_{i}(\tau_{\sigma },\tau_{ɛ})\right|}^{2}\text{d}\tau_{\sigma }\text{d}\tau_{ɛ}\\ & =\frac{1}{N}\sum_{i=1}^{N}\iint {\left|T_{i}(ο,\upsilon )-X_{i}^{*}(ο,\upsilon )H(ο,\upsilon )\right|}^{2}\text{d}ο\text{d}\upsilon \end{array}$$

where *T_i_* is the Fourier transform of *t_i_*. If ${\hat{\text{X}}}_{i}^{\text{D}}$ is obtained by converting the vectors ${\hat{\text{x}}}_{i}$ into diagonal matrices, then the average squared error of Equation (28) becomes

(29) $$E=\left[E_{d}-{\hat{\text{h}}}^{+}\hat{\text{p}}-{\hat{\text{p}}}^{+}\hat{\text{h}}+{\text{h}}^{+}\hat{\text{M}}\hat{\text{h}}\right]$$

where

(30) $$E_{d}=\frac{1}{N}\sum_{i=1}^{N}({\text{t}}_{i}^{+}{\text{t}}_{i})$$

(31) $$\hat{\text{p}}=\frac{1}{N}\sum_{i=1}^{N}({\text{X}}_{i}^{\text{D}}{\text{t}}_{i})$$

(32) $$\hat{\text{M}}=\frac{1}{N}\sum_{i=1}^{N}({\text{X}}_{i}^{\text{D}*}{\text{X}}_{i}^{\text{D}})$$

Minimizing *E* in Equation (29) subject to the constraints in Equation (25) leads to the following MSE-SDF filter

(33) $${\hat{\text{h}}}_{\text{MSE}-\text{SDF}}={\hat{\text{M}}}^{-1}\hat{\text{p}}+{\hat{\text{M}}}^{-1}\hat{\text{X}}\left[{\left({\hat{\text{F}}}^{+}{\hat{\text{M}}}^{-1}\hat{\text{F}}\right)}^{-1}\right]\;\left[\hat{\text{c}}-{\hat{\text{F}}}^{+}{\hat{\text{M}}}^{-1}\hat{\text{p}}\right]$$

The MSE-SDF filter allows approximating arbitrary correlation shapes rather than zero shape implied in MACE filter design. This explicit control has two benefits [21]. First, correlation shapes can be selected with the linear/nonlinear postprocessing in mind and second, those correlation shapes that lead to better filter design can be used instead of simply minimizing the average correlation energy.

## 7. Maximum Average Correlation Height (MACH) Filter

The primary objective of the correlation filters is to achieve distortion-tolerant recognition of objects in the presence of clutter. This problem is easier to solve for in-plane rotations and scale changes. However, the prevalent method for handling out-of-plane distortions is to use a training set of representative views of the object. Traditionally, in the design of SDF-type correlation filters, linear constraints are imposed on the training images to yield a known value at specific locations in the correlation plane. However, placing such constraints in the correlation plane satisfies conditions only at isolated points in the image space but does not explicitly control the filter's ability to generalize over the entire domain of the training images. Various filters exhibit different levels of distortion tolerance even with the same training set and constraints.

The MACH filter adopts a statistical approach for filter design [22,23]. In addition to yielding sharp peaks and being computationally simple, this filter offers improved distortion tolerance. The reason lies in the fact that training images are not treated as deterministic representations of the object but as samples of a class whose characteristic parameters should be used in encoding the filter.

It is assumed that the training set consists of N images, and that each image of size ρ_1_ × ρ_2_ contains ρ *=* ρ_1_ρ_2_ pixels. The *i*-th training image for the target class is denoted by *x_i_*(*m*,*n*) in the spatial domain, which is represented in the frequency domain by a ρ × 1 vector **x***_i_*, obtained by lexicographically reordering its two-dimensional discrete Fourier transform, *X_i_*(*k*,*l*). The Fourier domain filter is denoted by the ρ × 1 vector **h**. The two-dimensional filter *H*(*k*,*l*) is obtained by rearranging **h** into a two-dimensional image. In this paper, matrices are denoted by uppercase bold-face and vectors by lowercase bold-face characters. The correlation of the *i*-th training image and the filter can be expressed in the frequency domain as

(34) $${\text{g}}_{i}={\text{X}}_{i}\text{h}$$

where **X***_i_* is a ρ × ρ diagonal matrix containing the elements of **x***_i_*. Here, **g***_i_* denotes the discrete Fourier transform of the *i*-th correlation output. The deviation in the shape of the correlation plane with respect to some ideal shape vector **f** is quantified by the average squared error (ASE), defined as

(35) $$\text{ASE}=\frac{1}{N}\sum_{i=1}^{N}{\left({\text{g}}_{i}-\text{f}\right)}^{+}({\text{g}}_{i}-\text{f})$$

Thus, ASE is a measure of distortion with respect to reference shape **f**, which can be chosen as desired.

In fact, the shape vector **f** can be treated as a free parameter in the distortion minimization problem. In the design of MSE-SDF [21], **f** is specified as Gaussian or ring-like shapes in order to sculpt the correlation surface into these forms. In MACH, the choice of **f** is such that it causes least variation among the correlation planes and offers minimum ASE. To find the optimum shape **f**_opt_, the gradient of ASE with respect to **f** is set to zero, given by

(36) $$\nabla_{\text{f}}(\text{ASE})=\frac{2}{N}\sum_{i=1}^{N}({\text{g}}_{i}-\text{f})=0$$

or,

(37) $${\text{f}}_{\text{opt}}=\frac{1}{N}\sum_{i=1}^{N}{\text{g}}_{i}=\bar{\text{g}}$$

where

(38) $$\bar{\text{g}}=\frac{1}{N}\sum_{i=1}^{N}{\text{X}}_{i}\text{h}=\text{Mh}$$

is the average correlation plane and $\text{M}=(1/N)\sum_{i=1}^{N}{\text{X}}_{i}$ is the average training image expressed as a diagonal matrix. Thus, among all possible reference shapes, the average correlation plane $\bar{\text{g}}$ offers the smallest possible ASE and the least distortion (in the squared error sense) among the correlation planes.

Substituting $\text{f}=\bar{\text{g}}$ in the ASE expression, the average similarity measure (ASM) is obtained as

(39) $$\begin{array}{cc} \text{ASM} & =\frac{1}{N}\sum_{i=1}^{N}{\left({\text{g}}_{i}-\bar{\text{g}}\right)}^{+}({\text{g}}_{i}-\bar{\text{g}})\\ & =\frac{1}{N}\sum_{i=1}^{N}{\left({\text{X}}_{i}\text{h}-\text{Mh}\right)}^{+}({\text{X}}_{i}\text{h}-\text{Mh})\\ & ={\text{h}}^{+}\left[\sum_{i=1}^{N}{\left({\text{X}}_{i}-\text{M}\right)}^{*}({\text{X}}_{i}-\text{M})\right]\text{h}\\ & ={\text{h}}^{+}{\text{S}}_{x}\text{h} \end{array}$$

where

(40) $${\text{S}}_{x}=\sum_{i=1}^{N}{\left({\text{X}}_{i}-\text{M}\right)}^{*}({\text{X}}_{i}-\text{M})$$

is a diagonal matrix measuring the similarity of the training images to the class mean in the frequency domain. For example, if all training images are identical, then **S***_x_* would be an all-zero matrix. From Parseval's theorem, it is easy to show that the average squared distance from the correlation planes to their mean is the same as that defined by Equation (39) in the frequency domain [22].

The ASM is one possible metric for distortion since it represents the average deviation of the correlation planes from the mean correlation shape, $\bar{\text{g}}$. It is also a measure of the compactness of the class. If filter **h** is viewed as a linear transform, then ASM measures the distances of the training images from the class center under this transform. Minimizing ASM, therefore, leads to a compact set of correlation planes that resemble each other and exhibit the least possible variations. The distortions of the object in the input plane are represented by the training images, **x***_i_*. These distortions are reflected in the output as variations in the structure and shape of the corresponding correlation planes, **g***_i_*, and are quantified by ASM. If the filter successfully reduces the distortions, then distorted input images should yield similar output planes, leading to a small value of ASM. Conversely, if ASM is minimum and it is well shaped by design, then all true-class correlation planes are expected to resemble $\bar{\text{g}}$ and to exhibit well-shaped structures.

The MACH filter relaxes the correlation peak constraints and maximizes the peak intensity of the average training image. The peak intensity of the average training image is $|\bar{g}(0,0){|}^{2}$ expressed as

(41) $${\left|\bar{g}(0,0)\right|}^{2}={\left|\frac{1}{N}\sum_{i=1}^{N}{\text{h}}^{+}{\text{x}}_{i}\right|}^{2}={\left|{\text{h}}^{+}\text{m}\right|}^{2}={\text{h}}^{+}\text{m}{\text{m}}^{+}\text{h}$$

where **m** is the Fourier transform of the average training image expressed as a vector.

Here, it is assumed without the loss of generality that the peak occurs at the origin of the correlation plane.

The smaller the value of ASM, the more invariant the response of the filter is. In other words, if ASM is small, then all true-class correlation planes are expected to resemble $\bar{\text{g}}$. Therefore, it is required by **h** to produce high correlation peak with the mean image while making ASM small. In addition, it is also required to obtain some degree of noise tolerance to reduce the output noise variance (ONV). For additive input noise, ONV = **h**^+^**Dh**, where **D** is the diagonal power spectral density matrix [23]. While practical noise may be multiplicative and more complicated than implied by simple additive noise, the simple additive noise model at least provides some robustness. The performance criterion used to optimize the MACH filter may be expressed as

(42) $$\begin{array}{cc} J(\text{h}) & =\frac{{\left(\text{Average peak height}\right)}^{2}}{\text{ASM}+\text{ONV}}\\ & =\frac{{\left|\bar{g}(0,0)\right|}^{2}}{\text{ASM}+\text{ONV}}\\ & =\frac{{\left|{\text{h}}^{+}\text{m}\right|}^{2}}{{\text{h}}^{+}\text{Sh}+{\text{h}}^{+}\text{Dh}}\\ & =\frac{{\text{h}}^{+}\text{m}{\text{m}}^{+}\text{h}}{{\text{h}}^{+}(\text{S}+\text{D})\text{h}} \end{array}$$

The optimum solution is found by setting the derivative of *J*(**h**) in Equation (42) with respect to **h** to zero and is given by [22,23]

(43) $$\text{h}={\left(\text{S}+\text{D}\right)}^{-1}\text{m}$$

The filter in Equation (43) is referred to as the MACH filter because it maximizes the height of the mean correlation peak relative to the expected distortions. For cases where an estimate of **D** is not available, the white noise covariance matrix is substituted for **D**, *i.e.*, **D** = *σ*^2^**I**, where **I** is a diagonal identity matrix. Hence the simplified MACH filter becomes

(44) $$\text{h}={\left(\text{S}+\sigma^{2}\text{I}\right)}^{-1}\text{m}$$

Replacing *σ*^2^ by another constant *γ*, the filter equation becomes

(45) $$\text{h}={\left(\text{S}+\gamma \text{I}\right)}^{-1}\text{m}$$

The robustness of the MACH filter is attributed to the inclusion of the ASM criterion, which reduces the filter sensitivity to distortions, and to the removal of hard constraints on the peak. The later fact enables the correlation planes to adjust to suitable values for optimizing the performance criterion. However, MACH filter can handle those distortions that are well represented in the training set.

The Fourier domain MACH filter obtained in Equation (45) may be converted to the 2D shape or size of the input training images expressed as *H*(*k*,*l*). A 2D test image *z*(*m*,*n*) is Fourier transformed to obtain *Z*(*k*,*l*) which is then correlated with the 2D filter in the Fourier domain using the expression

(46) $$G(k,l)=Z(k,l)H^{*}(k,l)$$

The output spatial domain correlation is obtained by applying the inverse Fourier transform operation to Equation (46) and recording the intensity, given by

(47) $$g(m,n)={\left|{ℑ}^{-1}\left[G(k,l)\right]\right|}^{2}$$

## 8. Extended MACH (EMACH) Filter

The average training image used in the MACH filter design is good in representing the average behavior of the desired class, but it fails to capture the finer details of the desired class [24,25]. In fact, the average of training images sometimes looks like a clutter image. Thus, the MACH filter may be inadequate in discriminating the desired class from the clutter, leading to increased false alarm rate. The extended MACH (EMACH) filter is aimed at improving this clutter rejection capability.

The MACH filter is designed to maximize the intensity of the average correlation output at the origin due to training images. The average of correlation peaks is the correlation output due to the average training image. It also maximizes the similarity between the average training image correlation output and those outputs due to all training images from the desired class. Thus, the MACH filter forces all images from the desired class to follow the behavior of the average training image from that class. The MACH filter relies heavily on the mean training image. It amplifies the high-energy (usually low-frequency) components, and at the same time, attenuates the low-energy (usually high-frequency) components of the training set. Thus, by using the mean image **m** as the only example that represents all training images, a filter may be obtained that does not capture the finer details of the training images. Therefore, this filter may fail to discriminate the desired class from the clutter. The MACH filters possess attributes that may lead to detect clutter images as targets. One such attribute is that all training images follow the same behavior as the average training image. However, the average training image is not necessarily a good representative of the desired class.

To control the relative contribution of the desired class training images as well as their average, a new metric, called all image correlation height (AICH) [25], is introduced and defined as

(48) $$\begin{array}{cc} \text{AICH} & =\frac{1}{N}\sum_{i=1}^{N}{\left[{\text{h}}^{+}\left({\text{x}}_{i}-\beta \text{m}\right)\right]}^{2}\\ & =\frac{1}{N}\sum_{i=1}^{N}{\left({\text{h}}^{+}{\text{x}}_{i}-\beta {\text{h}}^{+}\text{m}\right)}^{2} \end{array}$$

where *β* is a parameter that takes a value between 0 and 1 and governs the relative significance of the average training image in the filter design. By controlling *β*, the designed filter is prevented from being overwhelmed by the biased treatment of the low-frequency components represented by the average image. Here, the AICH must be optimized and to be able to do that, Equation (48) may be rewritten as

(49) $$\begin{array}{cc} \text{AICH} & =\frac{1}{N}\sum_{i=1}^{N}\left({\text{h}}^{+}{\text{x}}_{i}-\beta {\text{h}}^{+}\text{m}\right){\left({\text{h}}^{+}{\text{x}}_{i}-\beta {\text{h}}^{+}\text{m}\right)}^{+}\\ & ={\text{h}}^{+}\left[\frac{1}{N}\sum_{i=1}^{N}\left({\text{x}}_{i}-\beta \text{m}\right){\left({\text{x}}_{i}-\beta \text{m}\right)}^{+}\right]\text{h}\\ & ={\text{h}}^{+}{\text{C}}_{x}^{\beta }\text{h} \end{array}$$

where

(50) $${\text{C}}_{x}^{\beta }=\frac{1}{N}\sum_{i=1}^{N}\left({\text{x}}_{i}-\beta \text{m}\right){\left({\text{x}}_{i}-\beta \text{m}\right)}^{+}$$

Thus, AICH can be described as the average of the correlation peak intensities of *N* exemplars where the *i*-th exemplar (x_i_ − *β*m) is the *i*-th training image with part of the mean subtracted. Hence, it is desirable for all images in the training set to follow these exemplars' behavior. This can be done by forcing every image in the training set **x***_i_* to have a similar correlation output plane to an ideal correlation output shape **f**. To find the **f** that best matches all these exemplars' correlation output planes, its deviation from their correlation planes is minimized. This deviation can be quantified by ASE, defined as

(51) $$\text{ASE}=\frac{1}{N}\sum_{i=1}^{N}{\left({\text{g}}_{i}-\text{f}\right)}^{+}\left({\text{g}}_{i}-\text{f}\right)$$

where,

(52) $${\text{g}}_{i}=({\text{X}}_{i}-\beta \text{M}){\text{h}}^{*}$$

In Equation (52), the superscript * represents the complex conjugate operation. To find the optimum shape vector **f**_opt_, the gradient of ASE with respect to **f** is set to zero, yielding

(53) $$\begin{array}{cc} {\text{f}}_{\text{opt}} & =\frac{1}{N}{\text{g}}_{i}\\ & =\frac{1}{N}\sum_{i=1}^{N}({\text{X}}_{i}-\beta \text{M}){\text{h}}^{*}\\ & =\left(1-\beta \right)\text{M}{\text{h}}^{*} \end{array}$$

The ASM is modified such that it measures the dissimilarity of the training images to (1 − *β*)Mh*. This new measure is called as the modified ASM (MASM), given by

(54) $$\begin{array}{cc} \text{MASM} & =\frac{1}{N}\sum_{i=1}^{N}{\left[{\text{X}}_{i}{\text{h}}^{*}-(1-\beta )\text{M}{\text{h}}^{*}\right]}^{+}\left[{\text{X}}_{i}{\text{h}}^{*}-(1-\beta )\text{M}{\text{h}}^{*}\right]\\ & ={\text{h}}^{\prime }\left\{\frac{1}{N}\sum_{i=1}^{N}{\left[{\text{X}}_{i}-(1-\beta )\text{M}\right]}^{*}\left[{\text{X}}_{i}-(1-\beta )\text{M}\right]\right\}{\text{h}}^{*}\\ & ={\text{h}}^{\prime }{\text{S}}_{x}^{\beta }{\text{h}}^{*}\\ & ={\text{h}}^{+}{\text{S}}_{x}^{\beta }\text{h} \end{array}$$

where the superscript' represents the transpose operation and where it is considered that MASM is real in deriving the last equality in Equation (54). The diagonal matrix ${\text{S}}_{x}^{\beta }$ is given by

(55) $${\text{S}}_{x}^{\beta }=\frac{1}{N}\sum_{i=1}^{N}{\left[{\text{X}}_{i}-(1-\beta )\text{M}\right]}^{*}\left[{\text{X}}_{i}-(1-\beta )\text{M}\right]$$

The ASM is a good measure for distortion tolerance; however, it lacks some discrimination capability that explains part of the MACH filter's inability to reject some clutter images. On the other hand, the MASM measure captures finer details of the training set that makes the EMACH filter more sensitive against clutter.

By maximizing the AICH and minimizing the MASM while controlling the parameter *β*, it is expected to explicitly keep a balance between the distortion tolerance and clutter rejection performance. Therefore, it is necessary to optimize the following new criterion

(56) $$J^{\beta }(\text{h})=\frac{\text{AICH}}{{\text{h}}^{+}\gamma \text{Ih}+{\text{h}}^{+}{\text{S}}_{x}^{\beta }\text{h}}=\frac{{\text{h}}^{+}{\text{C}}_{x}^{\beta }\text{h}}{{\text{h}}^{+}(\gamma \text{I}+{\text{S}}_{x}^{\beta })\text{h}}$$

where **h**^+^ *γ***Ih** is the ONV term assuming an additive white noise with variance *γ*. The ONV helps to maintain noise tolerance when *β* increases, especially, at those low energy components. By maximizing the preceding criterion, the following condition is obtained for the EMACH filter

(57) $${\left(\gamma \text{I}+{\text{S}}_{x}^{\beta }\right)}^{-1}{\text{C}}_{x}^{\beta }\text{h}=\lambda \text{h}$$

where *λ* is a scalar identical to *J^β^*(**h**). Thus, **h** must be an eigenvector of ${\left(\gamma \text{I}+{\text{S}}_{x}^{\beta }\right)}^{-1}{\text{C}}_{x}^{\beta }$ with the corresponding eigenvalue *λ*. Since *λ* is identical to *J^β^*(**h**), **h** should be the eigenvector that corresponds to the maximum eigenvalue. The other eigenvectors corresponding to the other nonzero eigenvalues provide smaller *J^β^*(**h**) values. However, they may provide better discriminatory performance, as *β* is not known *a priori*. So the EMACH filter may be expressed as

(58) $$\text{h}=\text{Dominant eigenvector}\left\{{\left(\gamma \text{I}+{\text{S}}_{x}^{\beta }\right)}^{-1}{\text{C}}_{x}^{\beta }\right\}$$

## 9. Distance Classifier Correlation Filter (DCCF)

This is a correlation-based distance classifier scheme for recognition and classification of multiple similar or dissimilar objects. The underlying theory uses shift-invariant filters to compute distances between the input image and ideal references under an optimum transformation. The two ideas of relaxing the constraints on the correlation values at the origin and looking at the entire correlation plane rather than just the peak value led to the development of distance classifier correlation filters DCCFs [26–28]. The DCCF formulation can be used with any number of classes.

In the DCCF design, a global transformation is determined such that the transformed images from the same class are close to each other, whereas transformed images from different classes are separated from each other. This global transform leads to one correlation filter for each class. The use of these correlation filters is similar to the use of other correlation filters except in the final step. The test image is correlated with the correlation filter, and the resulting correlation peak is determined. This correlation-peak value is used to determine the distance of the test image to this class. Distances of the test image to all classes are determined and the class yielding the smallest distance is chosen. This paradigm allows for the relaxation of the correlation output constraints and the use of the entire correlation output.

It is important to realize that the use of DCCFs is similar to the use of other correlation filters. This means that the correlation peaks move by the same amount corresponding to the shift in the input, *i.e.*, this is a shift-invariant operation. The DCCF concept has demonstrated promising performance on both infrared as well as synthetic aperture radar imagery [28]. Test results show that DCCFs outperform other correlation filters in recognizing targets while rejecting noise, clutter, and other confusing objects.

It is assumed that the training images are segmented and registered at a desired point. Fourier transform of an image *x*(*m*,*n*) of size ρ_1_ × ρ_2_ containing ρ *=* ρ_1_ρ_2_ pixels can be expressed as a ρ *×* 1 dimensional column vector **x** or as a ρ × ρ diagonal matrix **X** with the elements of **x** as its diagonal elements, *i.e.*, diagonal {**x**} = **X**. Sometimes, the same quantity may be expressed both as a vector, say **m***_x_*, and as a diagonal matrix **M***_x_*. This implies that **Hm***_x_* and **M***_x_***h** are equivalent.

The distance classifier uses a global transform denoted by **H** to separate the classes maximally while making them as compact as possible. For shift invariance, this transform matrix must be diagonal in the frequency domain. Multiplication of a vector **x** by a diagonal matrix **H** is equivalent to multiplying *X*(*k*,*l*) by *H*(*k*,*l*).

Here, a general *C*-class distance-classifier problem is analyzed by assuming that the peak correlation values are as different as possible for each of the classes although hard constraints are not used to enforce this. In addition, for each class, the correlation planes or their inverse Fourier transforms should be almost similar to the transformed ideal reference shape for this class. The correlation peaks are most likely at the origin for the registered training images but can occur elsewhere in the test cases depending on the location of the target.

Assume **x***_ik_* is the ρ-dimensional column vector containing the Fourier transform of the *i-*th image of the *k-*th class, 1 ≤ *i* ≤ *N* and 1 ≤ *k* ≤ *C*, where each class contains *N* training images. If **m***_k_* is the mean Fourier transform of class *k*, then

(59) $$\begin{array}{cc} {\text{m}}_{k}=\frac{1}{N}\sum_{i=1}^{N}{\text{x}}_{ik}, & 1\leq k\leq C \end{array}$$

The correlation peak at the origin between the mean training image from the *k-*th class and the filter **h** is given by ${\text{m}}_{k}^{+}\text{h}$. Thus, the overall mean Fourier transform of the entire training set becomes

(60) $$\text{m}=\frac{1}{C}\sum_{k=1}^{C}{\text{m}}_{k}$$

The correlation peak of the overall mean image **m** with the filter **h** is given by

(61) $${\text{m}}^{+}\text{h}=\frac{1}{C}\sum_{k=1}^{C}{\text{m}}_{k}^{+}\text{h}$$

which represents the overall average of the origin values of all correlation planes.

If the transformation **h** makes the in-class correlation planes similar, then the in-class peak values should be similar to each other and to their mean. Thus, to make the interclass separation between the correlation peaks large, the mean peak values of the classes are made as different as possible. Although several possible criteria might achieve this objective, the approach here is to increase the distance of all classes from the central mean. Toward this end, the following distance measure, called class separation, has been formulated

(62) $$\begin{array}{cc} A(\text{h}) & =\frac{1}{C}\sum_{k=1}^{C}{\left|{\text{m}}_{k}^{+}\text{h}-{\text{m}}^{+}\text{h}\right|}^{2}\\ & =\frac{1}{C}\sum_{k=1}^{C}{\text{h}}^{+}(\text{m}-{\text{m}}_{k}){\left(\text{m}-{\text{m}}_{k}\right)}^{+}\text{h}\\ & ={\text{h}}^{+}\text{Wh} \end{array}$$

where

(63) $$\text{W}=\frac{1}{C}\sum_{k=1}^{C}(\text{m}-{\text{m}}_{k}){\left(\text{m}-{\text{m}}_{k}\right)}^{+}$$

is a ρ × ρ, full (*i.e.*, non-diagonal) matrix of rank less than or equal to (*C*-1). The rank of **W** is less than or equal to (*C*-1), because it is obtained by the addition of *C* outer products of vectors (**m** − **m***_k_*), but these *C* vectors add up to a zero vector. If *A*(**h**) of Equation (62) is maximized, the class mean correlation peaks $\left({\text{m}}_{k}^{+}\text{h}\right)$ will differ significantly. It is also desired that the distance of transformed inputs to their average be small. This distance *B*(**h**), which measures the compactness of each class, is the same as ASM defined for each class as

(64) $$\begin{array}{cc} {\text{ASM}}_{k}=\frac{1}{N}\sum_{i=1}^{N}{\left|{\text{g}}_{ik}-{\bar{\text{g}}}_{k}\right|}^{2} & 1\leq k\leq C \end{array}$$

where

(65) $$\begin{array}{c} {\text{g}}_{ik}={\text{X}}_{ik}{\text{h}}^{*}\\ {\bar{\text{g}}}_{k}={\text{M}}_{k}{\text{h}}^{*} \end{array}$$

are the Fourier transforms of the correlation outputs due to the *i-*th training image **x***_ik_* and the average training image **m***_k_*, respectively from class *k*. Note that **X***_ik_* and **M***_k_* are diagonal matrices with **x***_ik_* and **m***_k_* along the diagonal. The ASM is a measure of the similarity of the training images of a class to their mean and hence a measure of the compactness of the class after transformation by **H**. Using Equations (64) and (65), ASM*_k_* can be rewritten as

(66) $$\begin{array}{cc} {\text{ASM}}_{k} & =\frac{1}{N}\sum_{i=1}^{N}{\left({\text{X}}_{ik}{\text{h}}^{*}-{\text{M}}_{k}{\text{h}}^{*}\right)}^{+}({\text{X}}_{ik}{\text{h}}^{*}-{\text{M}}_{k}{\text{h}}^{*})\\ & ={\text{h}}^{\prime }\left[\frac{1}{N}\sum_{i=1}^{N}{\left({\text{X}}_{ik}-{\text{M}}_{k}\right)}^{*}({\text{X}}_{ik}-{\text{M}}_{k})\right]{\text{h}}^{*}\\ & ={\text{h}}^{+}{\text{S}}_{k}\text{h} \end{array}$$

where

(67) $${\text{S}}_{k}=\frac{1}{N}\sum_{i=1}^{N}{\left({\text{X}}_{ik}-{\text{M}}_{k}\right)}^{*}({\text{X}}_{ik}-{\text{M}}_{k})$$

In Equation (67), **S***_k_* is a ρ × ρ diagonal matrix where each training image contains ρ pixels. The overall ASM for *C* classes is defined as

(68) $$\text{ASM}=B(\text{h})=\frac{1}{N}\sum_{k=1}^{C}{\text{h}}^{+}{\text{S}}_{k}\text{h}={\text{h}}^{+}\text{Sh}$$

where

(69) $$\text{S}=\frac{1}{C}\sum_{k=1}^{C}{\text{S}}_{k}$$

To make the in-class metric *B*(**h**) small and to make the inter-class distance metric *A*(**h**) large, the filter **h** is designed to maximize the ratio

(70) $$J(\text{h})=\frac{A(\text{h})}{B(\text{h})}=\frac{{\text{h}}^{+}\text{Wh}}{{\text{h}}^{+}\text{Sh}}$$

with respect to **h**. The filter **h** that maximizes *J*(**h**) in Equation (70) is the eigenvector of **S**^−1^**W** with the largest eigenvalue [28]. Because **W** is a non-diagonal matrix of rank less than or equal to (*C*-1), finding the dominant eigenvector of **S**^−1^**W** requires a special algorithm when the training images, and thus the desired filter is of larger size [28]. When *J*(**h**) is maximum, the correlation shape produced by an input image is expected to be similar to the mean shape for its true class with a peak value different from the average peak value of any other class. The DCCF filter may be expressed as

(71) $$\text{h}=\text{Dominant eigenvector}\left\{{\text{S}}^{-1}\text{W}\right\}$$

The DCCF is the first technique proposed for shift-invariant transform-domain distance calculations with a correlator and that are specifically designed to accommodate multiple targets at different locations in the same image. This filter deals with the entire correlation plane and not just one point at the origin. It transforms the input image into a new space in which the distance of a test input from the classes is computed. Given a test input **z**, the distances *d_k_* between the transformed input and the ideal shape for class *k* is computed as

(72) $$\begin{array}{cc} d_{k} & ={\left|{\text{H}}^{*}\text{z}-{\text{H}}^{*}{\text{m}}_{k}\right|}^{2}\\ & ={\left|{\text{H}}^{*}\text{z}\right|}^{2}+{\left|{\text{H}}^{*}{\text{m}}_{k}\right|}^{2}-2ℜ\{{\text{z}}^{+}\text{H}{\text{H}}^{*}{\text{m}}_{k}\}\\ & =p+b_{k}-2ℜ\{{\text{z}}^{+}{\text{h}}_{k}\} \end{array}$$

In Equation (72), *p* = |H*z|^2^ is the energy (independent of the class) of the transformed input test image **z**; *b_k_* = |H*m*_k_*|^2^ is the energy (independent of the **z)** of the transformed mean of class *k* and **h***_k_* = HH*m*_k_* is viewed as the effective filter for class *k*. Because there are only *C* classes for which distances must be computed, only *C* such filters are required.

In general, the targets may be anywhere in the input image. For shift-invariant distance calculation the interest is in the smallest value of *d_k_* over all possible shifts of the target with respect to the class references. In Equation (72), because *p* and *b_k_* are both positive and independent of the position of the target, the smallest value of *d_k_* over all shifts is obtained when the third term (*i.e.*, **z**^+^**h***_k_***)** is as large as possible. Therefore, this term is chosen as the peak value for full cross correlation of **z** and **h***_k_*.

## 10. Polynomial DCCF (PDCCF)

Linear transformations such as the DCCF are attractive because of their optimality when the underlying statistics are Gaussian with equal covariances [29]. However, the DCCF uses transformations based on the second order statistics and does not capture higher-order statistics in images. Hence, it does not necessarily capture all of the discrimination information in some cases. Examples of such cases are encountered when signal dependent or multiplicative noises are present, and when inputs have non-Gaussian statistics. In non-Gaussian statistics cases, DCCF capabilities may be improved by applying different nonlinearities to the input image. By using nonlinear transformations, it may be possible to extract more useful information for discrimination. Thus, the classes that are not well separated in the original image space may become more separated in the nonlinearly mapped space. Also, point nonlinearities are preferred because they reduce the computational complexity because a simple nonlinearity is being applied to each point without considering all points in the neighborhood.

The polynomial DCCF (DCCF) extends the DCCF to include point nonlinear mappings of the input patterns [29]. Examples of such nonlinear mappings correspond to powers of pixels of the input images. Even though the resulting PDCCF system is not linear with respect to the input patterns, it is still linear in the kernel. This property allows frequency domain techniques for the design, analysis, and implementation of this filter. Another important property is that this system works on different powers of input image pixels, which corresponds to a multi-dimensional correlation operation and thus extends the linear DCCF classification optimization criterion to a nonlinear one, and no nonlinear optimization is involved. Moreover, the PDCCF system provides a new framework for combining different correlation filters, where each filter in the system is optimized jointly with other filters.

The PDCCF first maps the input image *x*(*m*,*n*) into *x^j^*(*m*,*n*) via point nonlinearities η*_j_*, where *j* = 1, 2, …, *n*. Thus *x^j^*(*m*,*n*) is related to *x*(*m*,*n*) through the following relationship

(73) $$\eta_{j}:x(m,n)\to x^{j}(m,n)$$

In Equation (73), all *x^j^*(*m*,*n*) are assumed to have the same size as the input *x*(*m*,*n*). Examples of such mappings include various powers, logarithms, cosines, *etc.* The nonlinearly mapped input image *x*(*m*,*n*) is transformed by the filter *h_j_*(*m*,*n*) built using *x^j^*(*m*,*n*). The overall distance is obtained by adding the distances resulting from the shift-invariant minimum mean squared error computations between every transformed image of the input and its respective ideal transformed reference. Those ideal transformed references are computed *a priori* by using the nonlinear functions η_1_, η_2_, …, and η*_n_* followed by the application of the filters *h*_1_(*m*,*n*), *h*_2_(*m*,*n*), …, and *h_n_*(*m*,*n*), respectively.

In the linear DCCF, the filtered image *g*(*m*,*n*) resulting from transformation of *x*^1^(*m*,*n*) by *h*_1_(*m*,*n*) can be written as

(74) $$g(m,n)=h_{1}(m,n)⊙x^{1}(m,n)$$

where, ⊙ denotes the crosscorrelation and *x*^1^(*m*,*n*) = *x*(*m*,*n*). By augmenting the input image with *x*^2^(*m*,*n*), another term can be added involving the crosscorrelation of the *x*^2^(*m*,*n*) with *h*_2_(*m*,*n*) to obtain an output transformed by the two-term PDCCF, as shown below.

(75) $$g(m,n)=h_{1}(m,n)⊙x^{1}(m,n)+h_{2}(m,n)⊙x^{2}(m,n)$$

Thus, the PDCCF has more terms at its disposal with which it can achieve better discrimination. Clearly, these new nonlinear versions of inputs are completely dependent on the original inputs, and in that sense no new information is being created. However, the new representations enable the correlation filters to provide better recognition. By continuing to add more terms such as *h_j_*(*m*,*n*)⊙*x^j^*(*m*,*n*), the *n*-term PDCCF can be obtained as

(76) $$g(m,n)=\sum_{j=1}^{n}h_{j}(m,n)⊙x^{j}(m,n)$$

If the focus is on the point-wise power nonlinearities for all η*_j_*'s the nonlinear mapping of the input, *x^j^*(*m*,*n*) can be defined as

(77) $$x^{j}(m,n)={\left[x(m,n)\right]}^{j}$$

where, *j* ∈ ℝ∞. The power nonlinearity plays an important role in SAR images as it can enhance the bright scatters or the overall contrast [29]. The filters *h_j_*(*m*,*n*) are computed jointly, and thus the advantages of the closed form solutions and nonlinear systems are combined and exploited. In the following analysis, Ψ represents the set of all powers used to construct a particular PDCCF. Although the nonlinearity is applied in the spatial domain, in the analyses to follow (e.g., filter formation, distance calculation, *etc.*) all the quantities are actually in Fourier domain. For example, **x** is a vector obtained by lexicographical rearrangement of the Fourier transform of *x*(*m*,*n*). All vectors and matrices with superscript *j* (e.g., **x***^j^* and **X***^j^*) represent these vectors and matrices (**x** and **X**) with each of their elements in the image (spatial) domain raised to the *j-*th power.

Assume ${\text{m}}_{k}^{j}$ is the mean of class *k* of the Fourier transforms of training images resulting from raising all their pixels to the *j*-th power and **h***_j_* is the filter built for images raised to the *j*-th power. Each of the Fourier transforms of the original image along with the Fourier transforms of its variations can be represented by use of a single block vector. Thus, the Fourier transform of the mean images of class *k* after augmentation becomes

(78) $${\text{m}}_{k}=\left(\begin{array}{l} {\text{m}}_{k}^{1}\\ {\text{m}}_{k}^{2}\\ .\\ .\\ {\text{m}}_{k}^{n} \end{array}\right)$$

Further, the filters, **h**_1_, **h**_2_, … and **h***_n_* can be combined into one filter, **h** as follows

(79) $$\text{h}=\left(\begin{array}{l} {\text{h}}_{1}\\ {\text{h}}_{2}\\ .\\ .\\ {\text{h}}_{n} \end{array}\right)$$

With Equations (75), (78), and (79), the correlation peak at the origin produced in response to the mean image of class *k* is given by

(80) $${\bar{g}}_{k}(0,0)=\sum_{j=1}^{n}{\text{h}}_{j}^{+}{\text{m}}_{k}^{j}={\text{h}}^{+}{\text{m}}_{k}$$

The distance between the classes, after being augmented and then transformed by the filters **h**_1_, **h**_2_, …, and **h***_n_* can be expressed as

(81) $$\begin{array}{cc} A(\text{h}) & =\frac{1}{C}\sum_{k=1}^{C}{\left|\sum_{j=1}^{n}{\text{h}}_{j}^{+}{\text{m}}_{k}^{j}-\sum_{j=1}^{n}{\text{h}}_{j}^{+}{\text{m}}^{j}\right|}^{2}\\ & =\frac{1}{C}\sum_{k=1}^{C}{\left|{\text{h}}^{+}{\text{m}}_{k}-{\text{h}}^{+}\text{m}\right|}^{2}\\ & ={\text{h}}^{+}\text{Wh} \end{array}$$

where

(82) $$\text{W}=\frac{1}{C}\sum_{k=1}^{C}({\text{m}}_{k}-\text{m}){\left({\text{m}}_{k}-\text{m}\right)}^{+}$$

To separate the classes as much as possible, the filter is required to produce a large *A*(**h**). Simultaneously, the compactness of the classes needs to be increased after transformation by **h**_1_, **h**_2_, …, and **h***_n_*. The compactness is measured by the similarity of the training images of a class to their mean. It can be represented by the ASM of that class. In general, the ASM for class *k* is defined as

(83) $$\begin{array}{cc} {\text{ASM}}_{k}=\frac{1}{N}\sum_{i=1}^{N}{\left|{\text{g}}_{ik}-{\bar{\text{g}}}_{k}\right|}^{2} & 1\leq k\leq C \end{array}$$

where

(81) $$\begin{array}{c} {\text{g}}_{ik}={\text{X}}_{ik}^{j}{\text{h}}_{j}^{*}\\ {\bar{\text{g}}}_{k}={\text{M}}_{k}^{j}{\text{h}}_{j}^{*} \end{array}$$

are the Fourier transforms of the filtered images produced by the transform filters in response to the input image **x***^j^* and the mean image **m***_k_*, respectively. Thus, from Equations (83) and (84), ASM*_k_* can be written as

(85) $$\begin{array}{cc} {\text{ASM}}_{k} & =\frac{1}{N}\sum_{i=1}^{N}{\left|{\text{X}}_{ik}^{1}{\text{h}}_{1}^{*}+{\text{X}}_{ik}^{2}{\text{h}}_{2}^{*}+\ldots +{\text{X}}_{ik}^{n}{\text{h}}_{n}^{*}-{\text{M}}_{k}^{1}{\text{h}}_{1}^{*}-{\text{M}}_{k}^{2}{\text{h}}_{2}^{*}-...-{\text{M}}_{k}^{2}{\text{h}}_{n}^{*}\right|}^{2}\\ & =\frac{1}{N}\sum_{i=1}^{N}{\left[({\text{X}}_{ik}^{1}-{\text{M}}_{k}^{1}){\text{h}}_{1}^{*}+({\text{X}}_{ik}^{2}-{\text{M}}_{k}^{2}){\text{h}}_{2}^{*}+\ldots +({\text{X}}_{ik}^{n}-{\text{M}}_{k}^{n}){\text{h}}_{n}^{*}\right]}^{+}\left[({\text{X}}_{ik}^{1}-{\text{M}}_{k}^{1}){\text{h}}_{1}^{*}+({\text{X}}_{ik}^{2}-{\text{M}}_{k}^{2}){\text{h}}_{2}^{*}+\ldots +({\text{X}}_{ik}^{n}-{\text{M}}_{k}^{n}){\text{h}}_{n}^{*}\right]\\ & =\sum_{u=1}^{n}\sum_{v=1}^{n}{\text{h}}_{u}^{+}{\text{S}}_{\text{kuv}}{\text{h}}_{v}\\ & =({\text{h}}_{1}^{+}{\text{h}}_{2}^{+}...{\text{h}}_{n}^{+})\left[\begin{array}{llll} {\text{S}}_{k11} & {\text{S}}_{k12} & \ldots & {\text{S}}_{k1n}\\ {\text{S}}_{k21} & {\text{S}}_{k22} & \ldots & {\text{S}}_{k2n}\\ . & & & \\ . & & & \\ {\text{S}}_{kn1} & {\text{S}}_{kn2} & \ldots & {\text{S}}_{\text{knn}} \end{array}\right]\;\left(\begin{array}{l} {\text{h}}_{1}\\ {\text{h}}_{2}\\ .\\ .\\ {\text{h}}_{n} \end{array}\right)\\ & ={\text{h}}^{+}{\text{S}}_{k}\text{h} \end{array}$$

where

(86) $${\text{S}}_{\text{kuv}}=\left[\frac{1}{N}\sum_{i=1}^{N}{\text{X}}_{ik}^{u}{\left({\text{X}}_{ik}^{v}\right)}^{*}\right]-{\text{M}}_{k}^{u}{\left({\text{M}}_{k}^{v}\right)}^{*}$$

are all diagonal matrices and

(87) $${\text{S}}_{k}=\left[\begin{array}{llll} {\text{S}}_{k11} & {\text{S}}_{k12} & \ldots & {\text{S}}_{k1n}\\ {\text{S}}_{k21} & {\text{S}}_{k22} & \ldots & {\text{S}}_{k2n}\\ . & & & \\ . & & & \\ {\text{S}}_{kn1} & {\text{S}}_{kn2} & \ldots & {\text{S}}_{\text{knn}} \end{array}\right]$$

The overall ASM for *C* classes is then defined as

(88) $$\text{ASM}=B(\text{h})=\frac{1}{N}\sum_{k=1}^{C}{\text{h}}^{+}{\text{S}}_{k}\text{h}={\text{h}}^{+}\text{Sh}$$

where

(89) $$\text{S}=\frac{1}{C}\sum_{k=1}^{C}{\text{S}}_{k}$$

The filter **h** that maximizes the ratio *A*(**h**)/*B*(**h)**, is the dominant eigenvector of **S**^−1^**W** as in case of DCCF and given by

(90) $$\text{h}=\text{Dominant eigenvector}\left\{{\text{S}}^{-1}\text{W}\right\}$$

Given a test input **z**, the distances *d_k_* between the transformed input and the ideal shape for class *k* is computed by using MSE-then-total approach [30]. In this approach, *n* distances are computed for class *k*. The *j-*th distance, *d_jk_*, is defined as follows

(91) $$\begin{array}{cc} d_{jk}={\left|{\text{H}}_{j}^{*}{\text{z}}^{j}-{\text{H}}_{j}^{*}{\text{m}}_{k}^{j}\right|}^{2} & 1\leq j\leq n \end{array}$$

The distance *d_jk_* can be rewritten as

(92) $$d_{jk}=p_{j}+b_{jk}-2ℜ\{{\left({\text{z}}^{j}\right)}^{+}{\text{h}}_{jk}\}$$

where

(93) $$p_{j}={\left|{\text{H}}_{j}^{*}{\text{z}}^{j}\right|}^{2}$$

(94) $$b_{jk}={\left|{\text{H}}_{j}^{*}{\text{m}}_{k}^{j}\right|}^{2}$$

(95) $${\text{h}}_{jk}={\text{H}}_{j}{\text{H}}_{j}^{*}{\text{m}}_{k}^{j}$$

The inner products, shown as the third terms in Equation (92), are between the *j*-th variation of the input, **z***^j^* and the corresponding *j-*th filter, **h***_kj_*. The total distance d*_k_* to a class is then found by

(96) $$d_{k}=\sum_{j=1}^{n}d_{jk}$$

The input image is assigned to the class with the least total distance.

## 11. Target Tracking in FLIR Imagery

In general, tracking of a moving pattern/target requires recognizing and then locating the target in a scene, finding the target motion, understanding the direction of motion of the target, and then following that target as it moves through the sequence of image frames. The detection and tracking of desired targets in a real life image corrupted by noise, clutter, illumination and other three-dimensional (3D) artifacts, poses a very complex problem and demands sophisticated solutions using pattern recognition and motion estimation methods [31,32]. Things become more complicated if there are more than one target in the scene and simultaneous multiple targets tracking is required.

Forward-looking infrared (FLIR) images are frequently used in automatic target recognition (ATR) applications. It is challenging to detect and track targets in FLIR imagery. To detect independent moving objects in FLIR image sequences, the sensor properties have to be taken into account. Additional challenges are caused due to many important differences of FLIR images with visual sequences [33,34]. Many researchers have investigated various approaches for detection, recognition, classification and pose estimation of targets from FLIR images including both matched spatial filter (MSF) based correlators and joint transform correlators [33–40]. However, the application of MSFs or their variants (e.g., MACH, DCCF) for the FLIR imagery is very limited; although those have been used for the simulated and real synthetic aperture radar (SAR) and laser radar (LADAR) imagery [24,41–47].

In this section, three different algorithms are demonstrated for pattern recognition and tracking based on the combination of the detection and classification filters [48–51]:

- MACH filter-based detection and DCCF-based classification (MACH-DCCF)
- MACH filter-based detection and PDCCF-based classification (MACH-PDCCF)
- EMACH filter-based detection and PDCCF-based classification (EMACH-PDCCF)

The detection filters are trained by the target images of expected size and orientation variations with expected size of input scene. The classification filters are formulated with the expected size of target images and trained by the target images of expected size and orientation variations. The first step of the real time system is detection, which involves correlating the input scene with all detection filters (one for each desired or expected target class) and combining the correlation outputs. In the second step, a predefined number of ROIs having the expected size of target images are selected based on the regions having higher correlation peak values in the combined correlation output. To ensure that all desired or expected targets are included in the ROIs, the number of ROIs should be at least three times higher than the number of expected targets. Classification filters are then applied to these ROIs and target types along with clutters are identified based on a distance measure and a threshold. Moving target detection and tracking are accomplished by following this technique for all incoming image frames by applying the same filters.

**Figure 1.**
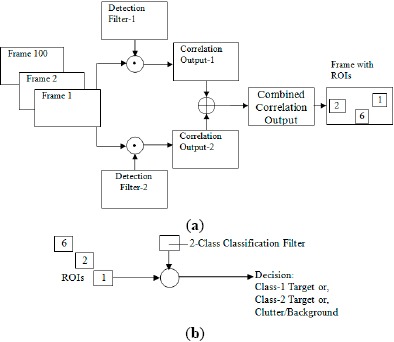
Schematic diagram of the proposed technique for (**a**) detection stage; and (**b**) classification stage.

Multiple detection filters and classification filters are formulated for each target based on different size ranges or aspect angles. All of the filters for different ranges can be applied simultaneously through the whole range of the image sequence and decision can be made based on the output of the filter corresponding to the highest correlation peak or minimum distance. However, in this illustration, one detection filter and one classification filter is used for each class of targets for a particular range. A block diagram of the method for real time pattern recognition and tracking is shown in Figure 1 for a two-class detection system.

### 11.1. Image Dataset

The FLIR image database used in this research is supplied by Army Missile Command (AMCOM). This image database has a total of 50 real life infrared video sequences, some of which contain a single target in the scene and some contain multiple targets. In general, the image sequences are closing sequences, *i.e.*, the targets become closer to the observer as the later frames appear in the scene. Thus, the size and signature of the targets change from the first frame to the last frame. Moreover, as the targets move, there are changes in the targets' orientations from one frame to the next. The database is also associated with ground truth data files containing the list of targets at each frame of each image sequence and their size and location in the frame. Among the 50 sequences, the proposed techniques have been applied for several single and multiple targets image sequences and the techniques are still being tested on other remaining sequences. For this paper, the analysis on 4 single target sequences (L1415, L2018, L2312, M1406), 2 two-target sequences (L1701, L1911) and 1 three-target sequence (L1618) have been reported.

### 11.2. Single-Target Image Sequences

At first, consider Sequence L2018, which has the highest difficulty level among the selected single target image sequences. In this sequence, there are a total of 448 frames, each of which contains the same target (tank1). The size of this target in the first frame is 3 × 4 pixels whereas that in the final frame is 12 × 23 pixels. For effective detection and classification, proper selection of the training images is important. It is obvious that a single detection or classification filter obtained by exploiting the training images from this large variation of sizes may lose its selectivity. So, for this particular sequence, two different detection filters (MACH or EMACH) are formed for the target to use in two ranges of the image frames depending on the size of the target.

**Figure 2.**
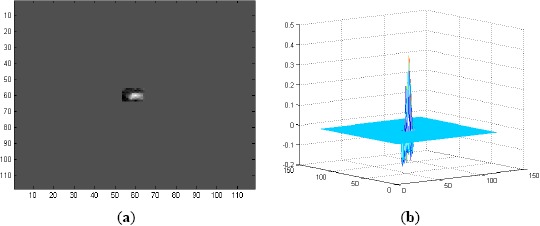
(**a**) A full size (118 × 118-pixel) training image for detection filters created from the target patch of Frame 100 of L2018; and (**b**) 3D mesh plot of (**a**).

**Figure 3.**
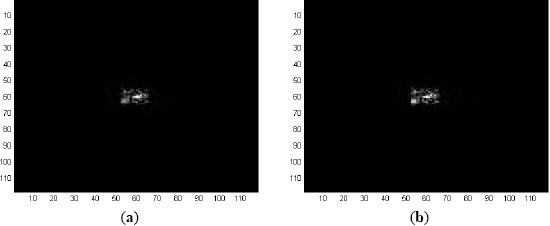
Spatial domain representation of Range-1 detection filters for the target (tank1) of Sequence L2018 (**a**) MACH filter; and (**b**) EMACH filter.

The first range detection filters are trained using the target patches taken from the first 200 frames at an interval of 5 frames (the 1st, 5th, 10th, 15th, …, 200th frames). The ground truth data files are used to read the coordinates and sizes of the targets and then to segment out the target patches from the original frames. Each of these patches is then mean-subtracted and normalized dividing by the RMS value of the mean-subtracted patch. Thereafter, each patch is placed at the center of a 118 × 118-pixel zero padded matrix to form a full size training image for the detection filters. A sample training image of this type for Sequence L2018 is shown in Figure 2. The spatial domain representations of the MACH filter and EMACH filter for Range-1, trained by the above mentioned training images, are shown in Figure 3a,b, respectively.

**Figure 4.**
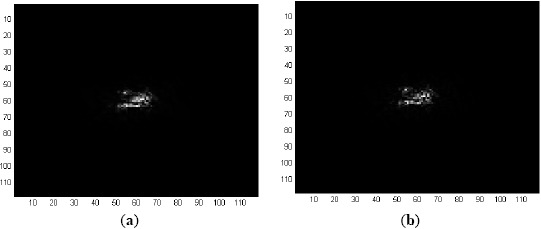
Spatial domain representation of Range-2 detection filters for the target (tank1) of Sequence L2018 (**a**) MACH filter; and (**b**) EMACH filter.

The detection filters for Range-2, to use from Frame 201 to Frame 448, are trained by the target patches taken from the Frames 201 to 300 at an interval of 5 frames. The corresponding Range-2 MACH and EMACH filters are shown in Figure 4a,b, respectively.

**Figure 5.**
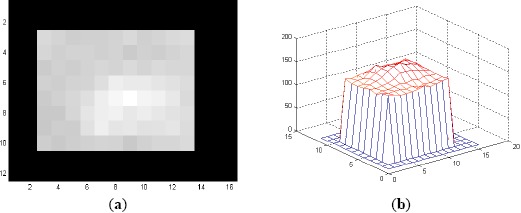
(**a**) A 12×16-pixel training image for classification filters created from the target patch of Frame 100 of L2018; and (**b**) 3D mesh plot of (a).

In general, the classification filters (DCCF or PDCCF) are also required to be formulated for different ranges of the targets for better selectivity. The sizes of these filters are usually chosen as the expected size of the targets in the corresponding range of image frames. Target patches are taken as before but they are not normalized for classification filters. Each patch is placed at the center of a zero padded background having the size of the classification filter. If the patch is larger than the filter size, it is truncated at the sides. For Sequence L2018, it is found that the classification filter (either DCCF or PDCCF) of 12 × 16 pixels trained by the target patches of Frame 1 to Frame 200 at an interval of 4 frames works well for almost all frames. A 12 × 16-pixel sample training image of tank1 of Sequence L2018 for classification filter is shown in Figure 5.

It is assumed that the single target sequences are known to have only one target of interest. Hence, in this work, single class (1-class) classification filters (DCCF, PDCCF) are formulated for each particular sequence using the corresponding target patches. Although the classification filters actually need to be formulated using at least two classes of training images, in this work, 1-class filters are formulated by using slight modification in the basic formula. However, these 1-class approximated filters have been found to work well in most cases.

The training data or parameters for all the four filters (MACH, EMACH, DCCF, PDCCF) used for the three algorithms for detecting and tracking the target (tank1) in Sequence L2018 are summarized in Table 1. The first column of the table represents the name of the target along with the number of frames for which the ground truth data is available for it. The second column shows the filter names with their range indices; and the third column shows the range of the actual image frames where a particular filter is applied. The fourth and fifth columns provide the considered range of the frames and the order or interval of the frames used to take the target patch for training. In Table 1, *γ* is the filter parameter for MACH; *β* and *γ* are the filter parameters for EMACH and Ψ is the set of nonlinear powers used for PDCCF formulation.

Filter formulations of the three other single target sequences (L1415, L2312 and M1406) are also similar to Sequence L2018. To overcome the size variation from the initial frames to the final frames, multiple filters of multiple range bins are required in some cases to apply at different ranges. The training data for these three single target sequences are depicted in Tables 2–4, respectively.

**Figure 6.**
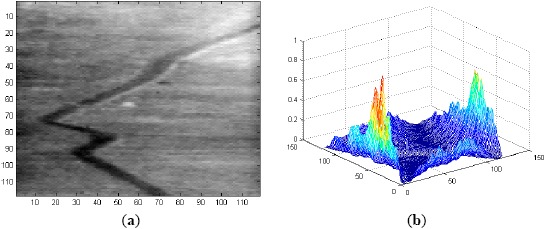
(**a**) Frame 1 of Sequence L2018; and (**b**) correlation output with the filter maximum average correlation height (MACH) Range-1.

**Figure 7.**
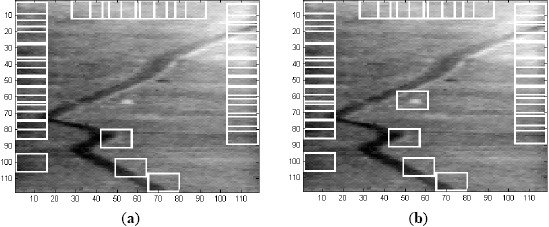
(**a**) 32 ROIs in Frame 1 of Sequence L2018; and (**b**) 33 ROIs in Frame 1 of Sequence L2018.

For analysis, consider the first frame (Frame 1) of Sequence L2018 shown in Figure 6a, apply Fourier transform to it and then correlate with the Fourier domain filter MACH Range-1 to obtain the correlation output of Figure 6b. It is obvious that the correlation output contains false peaks and high-energy diffractions at low frequency which make the actual correlation signal negligible. For this reason, it requires 33 ROIs to include the target of interest as shown in Figure 7b.

**Figure 8.**
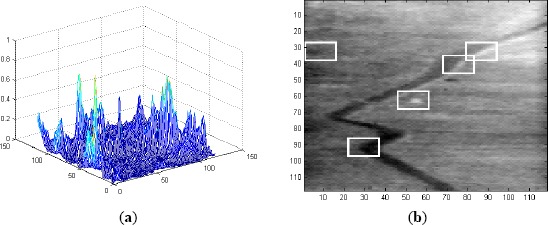
(**a**) Correlation output for Frame 1 of Sequence L2018 using MACH Range-1 filter along with notch filter; and (**b**) 5 ROIs in Frame 1.

To eliminate the strong low frequency components, a notch filter is used before applying the inverse Fourier transform operation, which actually suppresses the Fourier domain components along the axes to zero. The correlation output after applying this notch filter is shown in Figure 8a. Five ROIs in Frame 1, selected based on this correlation output, are shown in Figure 8b where the target (tank1) is included within these ROIs. This notch filter is used in the detection stage of each frame using either MACH or EMACH filter for all image sequences analyzed in this work. The detection results using EMACH filter are similar to MACH filter with or without the use of notch filter.

It is obvious that the detection and classification cannot be done correctly with detection filters alone. Because of the presence of multiple identical and different targets and clutters, the highest correlation peak or PSR value is not always guaranteed for the desired target. Therefore, classification filters are used for improved discrimination for the single target image sequences too. It is found that the 1-class classification filters work well in rejecting the clutters and backgrounds. However, to make a decision for identifying the target and rejecting the clutter the ROI having the least distance is considered as the potential target. If the distance to the ROI having the second minimum distance is not higher than the prescribed percentage of the least distance, then the ROI having the least distance is rejected as clutter or background. Since EMACH filter has improved clutter rejection capability, the application of EMACH filter in the detection stage facilitates in lowering the number of ROIs introduced into the classification stage. For this particular sequence (L2018), it is found in the simulation that 8 ROIs are required to include the target in almost all frames in the case of MACH filter, whereas 6 ROIs are sufficient with EMACH filter. However, for generality, 8 ROIs are also considered in case of EMACH filter for this sequence.

**Figure 9.**
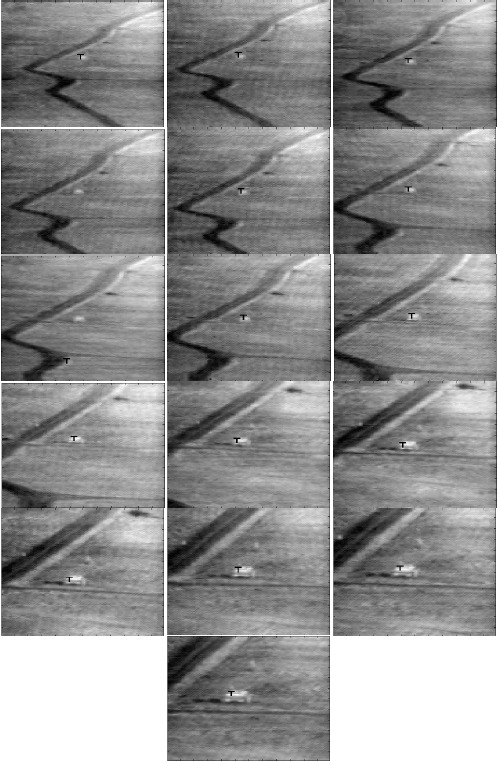
Target detection and classification results of Sequence L2018.

All single target sequences are tested by using the three developed algorithms: MACH-DCCF, MACH-PDCCF and EMACH-PDCCF. Some of the frames of Sequence L2018 showing the results after applying the EMACH-PDCCF algorithm are given in Figure 9. The tracking algorithm inserts a “T” mark at the locations of the detected targets (tank1) at each frame as shown in Figure 9. The number at the lower left corner of each frame indicates the frame number. In Figure 9, some sample frames are also included where the classification is incorrect or no decision is inferred. The detection and tracking results for all single target sequences are summarized for all the three algorithms in Table 5. In the threshold column of the table, a single value indicates that a single threshold is used for all ranges. From Table 5, it is observed that the poorest performance is achieved for Sequence L2018 among the four single target sequences presented. This may be attributed to the scene complexities and the drawback of using same classification filters through all the ranges of the sequence.

### 11.3. Two-Target Image Sequences

To assess the performance of the algorithms with two targets present in the scene, consider Sequences L1701 and L1911. The two targets in Sequence L1701 are Bradley and pickup. Out of 388 frames of this sequence, ground truth data for Bradley is available for 371 frames and that for pickup is available for 43 frames. The pickup disappears from the scene at Frame 31 and reappears at Frame 81 and again disappears at a later frame. The two targets, APC1 and tank1, in Sequence L1911 are present in all the 165 frames of the sequence. In these image sequences, the target sizes increase significantly from the first frame to the final frame. Therefore, different filters must be used for different ranges. For a particular range, two detection filters (MACH or EMACH) and a 2-class classification filter (DCCF or PDCCF) are required for a two-target sequence. Table 6 shows the training data/parameters selected for different filters for Sequence L1701 while Table 7 displays the same parameters for Sequence L1911. It may be mentioned that even if one target disappears after a few frames, both the detection filters and the 2-class classification filter are continued to apply to the remaining image frames; because in reality, it is not known whether any target is going out or coming back. This ensures the detection of a target that may reappear after a few frames.

**Figure 10.**
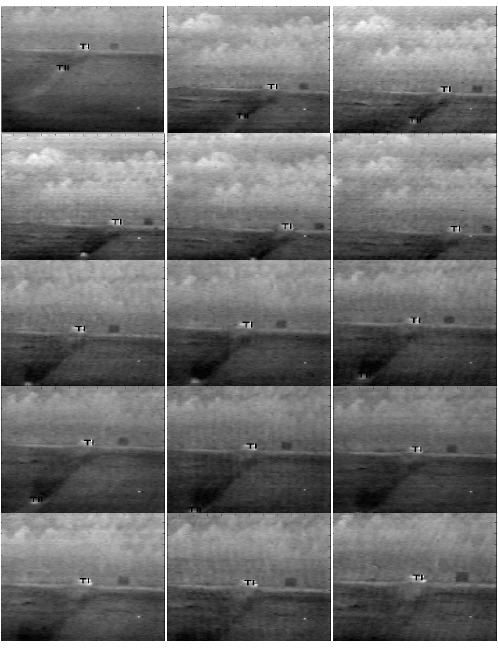

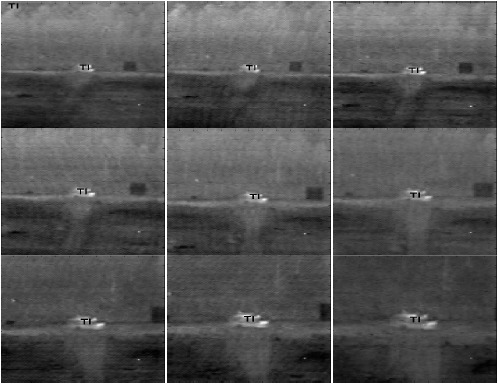
Target detection and classification results of Sequence L1701.

Using the detection and classification filters having the design parameters in Tables 6 and 7, all algorithms are tested for detection, classification and tracking of the objects in Sequences L1701 and L1911. The results obtained after applying the EMACH-PDCCF algorithm on Sequence L1701 are shown for some sample frames in Figure 10. The tracking algorithm inserts “T1” for the detected Class-1 type targets (Bradley) and “TII” for the detected Class-2 type targets (pickup) at their corresponding location in the image frame. The frame number is shown at the lower left corner of each frame displayed. From Figure 10, it is obvious that the tracking algorithm can successfully detect and classify the targets when they are present in the input scene. The complete tracking results for both the sequences are summarized in Table 8 for all algorithms. In the threshold column of the table, a single value indicates that a single threshold is used for all ranges. Otherwise, different threshold values in that column displays the thresholds used for various ranges of classification filters employed. It is observed that MACH-DCCF algorithm fails for Sequence L1911 in detecting and classifying the targets, and rejecting the clutters. On the other hand, EMACH-PDCCF algorithm provides the best results considering all the factors simultaneously, such as, required number of ROIs, percentage of successful detection and the total number of false alarms.

### 11.4. Three-Target Image Sequences

The three target images in Sequence L1618 are APC1, M60 and truck. Out of 300 frames in this sequence, ground truth data for APC1 is available for 291 frames, ground truth data for M60 is found for 101 frames and that for truck is found for 6 frames. The truck disappears from the sequence at Frame 18 and M60 disappears from the sequence at Frame 104. Like the other sequences, the size of the targets increases significantly from the first frame to the final frame. Thus, different filters are required for different ranges of this sequence. Assuming three expected targets in the scene for any particular range, three detection filters (MACH or EMACH) and a three-class classification filter (DCCF or PDCCF) are required for every frame in each range of this sequence. The training data/parameters for different filters, chosen for Sequence L1618 are displayed in Table 9.

**Figure 11.**
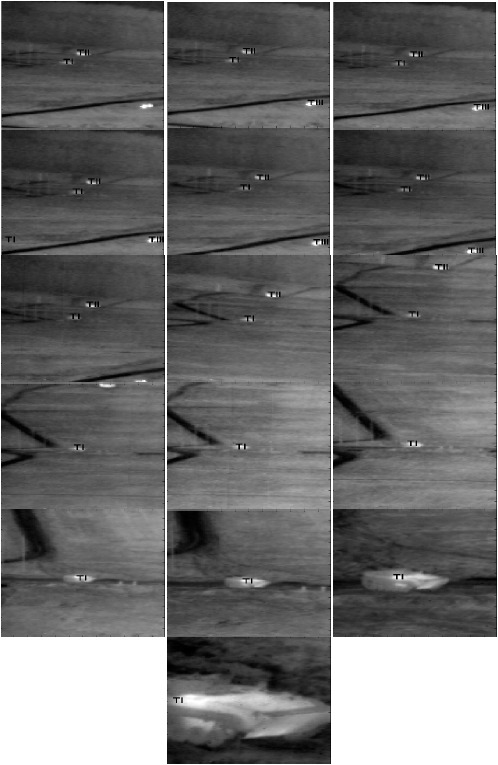
Target detection and classification results of Sequence L1618.

To evaluate the performance of all the algorithms for detection, clutter rejection, and classification as well as tracking of the three objects in three-target sequence, the designed detection and classification filters of Table 9 for different ranges are applied to Sequence L1618. The results obtained for the EMACH-PDCCF algorithm with Sequence L1618 are shown for some sample frames in Figure 11. The tracking algorithm places “T1” for the detected Class-1 type targets (APC1), “TII” for the detected Class-2 type targets (M60) and “TIII” for the detected Class-3 type targets (truck) at their corresponding locations in the frames. In Figure 11, the frame numbers are shown at the lower left corner of each frame. The complete tracking results for the sequence are summarized in Table 10 for all algorithms. In the threshold column of the table, a single value indicates that a single threshold is used for all ranges. Otherwise, different threshold values in that column displays the thresholds used for various ranges of classification filters employed.

## 12. Conclusions

Pattern recognition and tracking in FLIR imagery is a challenging problem due to various factors such as low resolution, low signal-to-noise ratio, different 3D orientations of the targets, effects of global motion, and close proximity with similar objects. In this paper, we reviewed the recent trends and advancements in distortion-invariant pattern recognition algorithms for single/multiple target detection and tracking in FLIR imagery using correlation filters. Each detection/tracking algorithm utilizes various properties of targets and image frames of a given FLIR sequence. Test results using real life FLIR image sequences are presented to verify the effectiveness of the filter-based pattern recognition and tracking techniques. Future work in this area would include a review of techniques beyond correlation that are particularly useful for high resolution targets. Also, development and inclusion of techniques with a dynamic update of the target model may be considered.

## Figures and Tables

**Table 1.** Training data for Sequence L2018.

**Target (No. of Frames)**	**Filter**	**Working Frame Range**	**Training Frames**		**Filter Size**	***β***	***γ***	**Ψ**

			**Range Taken**	**Interval Taken**				
tank1 (448)	MACH Range-1	1–200	1–200	5	118 × 118	-	0.1	-
	MACH Range-2	201–448	201–300	5	118 × 118	-	1.0	-
	EMACH Range-1	1–200	1–200	5	118 × 118	0.2	0.1	-
	EMACH Range-2	201–448	201–300	5	118 × 118	0.1	1.0	-

-	DCCF	1–448	1–200	4	12 × 16	-	-	-
-	PDCCF	1–448	1–200	4	12 × 16	-	-	1.0, 1.5, 2.0

**Table 2.** Training data for Sequence L1415.

**Target (No. of Frames)**	**Filter**	**Working Frame Range**	**Training frames**		**Filter Size**	***β***	***γ***	**Ψ**

			**Range Taken**	**Interval Taken**				
Mantruck (281)	MACH	1–281	1–100	5	118 × 118	-	1	-
	EMACH	1–281	1–100	5	118 × 118	0.1	1	-

-	DCCF	1–281	1–100	5	12 × 16	-	-	-
-	PDCCF	1–281	1–100	5	6 × 8	-	-	1.0, 1.5, 2.0

**Table 3.** Training data for Sequence L2312.

**Target (No. of Frames)**	**Filter**	**Working Frame Range**	**Training Frames**		**Filter Size**	***β***	***γ***	**Ψ**

			**Range Taken**	**Interval Taken**				
APC1 (368)	MACH	1–368	1–100	5	118 × 118	-	1	-
	EMACH	1–368	1–300	10	118 × 118	0.1	1	-

-	DCCF	1–368	1–300	10	12 × 16	-	-	-
-	PDCCF	1–368	1–300	10	8 × 12	-	-	1.0, 1.5, 2.0

**Table 4.** Training data for Sequence M1406.

**Target (No. of Frames)**	**Filter**	**Working Frame Range**	**Training Frames**		**Filter Size**	***β***	***γ***	**Ψ**

			**Range Taken**	**Interval Taken**				
Bradley (380)	MACH	1–380	1–100	5	118 × 118	-	1	-
	EMACH	1–380	1–100	5	118 × 118	0.1	1	-

-	DCCF	1–380	1–300	10	14 × 16	-	-	-
-	PDCCF Range-1	1–200	1–100	5	8 × 10	-	-	1.0, 1.5, 2.0
-	PDCCF Range-2	201–380	201–300	5	8 × 10	-	-	1.0, 1.5, 2.0

**Table 5.** Tracking results of single-target sequences.

**Seq. Name**	**Total Frames**	**No. of ROIs Taken**	**Threshold**	**Target Name**	**No. of Frames Target Present**	**No. of Frames Detected Correctly**	**Total No. of False Alarms**	**Percentage of Successful Detection**
MACH-DCCF Algorithm								

L1415	281	4	1.00	mantruck	281	281	0	100
L2018	448	8	0.99	tank1	448	357	36	80
L2312	368	4	1.00	APC1	368	366	2	99
M1406	380	4	1.00	Bradley	380	380	0	100

MACH-PDCCF Algorithm								

L1415	281	4	1.00	mantruck	281	263	9	94
L2018	448	8	0.99	tank1	448	371	37	83
L2312	368	4	1.00	APC1	368	368	0	100
M1406	380	4	1.00	Bradley	380	353	27	93

EMACH-PDCCF Algorithm								

L1415	281	4	1.00	mantruck	281	274	1	98
L2018	448	8	1.00	tank1	448	386	60	86
L2312	368	4	1.00	APC1	368	368	0	100
M1406	380	4	1.00	Bradley	380	354	26	93

**Table 6.** Training data for Sequence L1701.

**Target (No. of Frames)**	**Filter**	**Working Frame Range**	**Training Frames**		**Filter Size**	***β***	***γ***	**Ψ**

			**Range Taken**	**Interval Taken**				
Bradley (371)	MACH Range-1	1–100	1–100	5	118 × 118	-	1.0	-
	MACH Range-2	101–200	101–200	5	118 × 118	-	1.0	-
	MACH Range-3	201–300	201–300	5	118 × 118	-	0.1	-
	MACH Range-4	301–388	301–370	5	118 × 118		0.1	-
	EMACH Range-1	1–100	1–100	5	118 × 118	0.1	1.0	-
	EMACH Range-2	101–200	101–200	5	118 × 118	0.1	1.0	-
	EMACH Range-3	201–300	201–300	5	118 × 118	0.1	0.1	-
	EMACH Range-4	301–388	301–370	5	118 × 118	0.1	0.1	-

Pickup (43)	MACH Range-1	1–388	1–43	3	118 × 118		1.0	-
	EMACH Range-1	1–388	1–43	3	118 × 118	0.1	1.0	-

-	DCCF Range-1	1–100	1–100	5	6 × 8	-	-	-
-	DCCF Range-2	101–200	201–300	5	8 × 12	-	-	-
-	DCCF Range-3	201–300	201–300	5	10 × 18	-	-	-
-	DCCF Range-4	301–388	301–370	5	10 × 18	-	-	-
-	PDCCF Range-1	1–100	1–100	5	8 × 8	-	-	1.0, 1.5, 2.0
-	PDCCF Range-2	101–200	101–200	5	8 × 12	-	-	1.0, 1.5, 2.0
-	PDCCF Range-3	201–300	201–300	5	10 × 18	-	-	1.0, 1.5, 2.0
-	PDCCF Range-4	301–388	301–370	5	10 × 18	-	-	1.0, 1.5, 2.0

**Table 7.** Training data for Sequence L1911.

**Target (No. of Frames)**	**Filter**	**Working Frame Range**	**Training Frames**		**Filter Size**	***β***	***γ***	**Ψ**

			**Range Taken**	**Interval Taken**				
APC1 (165)	MACH Range-1	1:100	1:100	5	118 × 118	-	0.1	-
	MACH Range-2	101:165	101:150	5	118 × 118	-	0.1	-
	EMACH Range-1	1:100	1:100	5	118 × 118	0.1	0.1	-
	EMACH Range-2	101:165	101:150	5	118 × 118	0.1	0.1	-

tank1 (165)	MACH Range-1	1:100	1:100	5	118 × 118	-	0.1	-
	MACH Range-2	1:165	101:150	5	118 × 118	-	0.1	-
	EMACH Range-1	1:100	1:100	5	118 × 118	0.1	0.1	-
	EMACH Range-2	1:165	101:150	5	118 × 118	0.1	0.1	-

-	DCCF Range-1	1:100	1:100	5	8 × 16	-	-	-
-	DCCF Range-2	101:130	101:130	5	8 × 16	-	-	-
-	DCCF Range-3	131:165	131:160	5	16 × 36	-	-	-
-	PDCCF Range-1	1:130	1:100	5	8 × 16	-	-	1.0, 1.5, 2.0
-	PDCCF Range-2	1:130	1:100	5	8 × 16	-	-	1.0, 1.5, 2.0
-	PDCCF Range-3	131:165	131:160	5	16 × 36	-	-	1.0, 1.5, 2.0

**Table 8.** Tracking results of two-target sequences.

**Seq. Name**	**Total Frames**	**No. of ROIs Taken**	**Threshold**	**Target Name**	**No. of Frames Target Present**	**No. of Frames Detected Correctly**	**Total No. of False Alarms**	**Percentage of Successful Detection**
MACH-DCCF Algorithm								

L1701	388	6	0.70, 0.30, 0.05, 0.04	Bradley	388	230	248	59
				pickup	45	26	2	58

L1911	165	6	0.40	APC1	-	-	-	Fails
				Tank1	-	-	-	Fails

MACH-PDCCF Algorithm								

L1701	388	6	0.40, 0.05, 0.06, 0.06	Bradley	388	369	12	95
				pickup	45	28	1	62

L1911	165	6	0.40	APC1	165	152	0	92
				tank1	165	165	3	100

EMACH-PDCCF Algorithm								

L1701	388	4	0.40, 0.05, 0.06, 0.06	Bradley	388	369	9	95
				pickup	45	31	8	69

L1911	165	4	0.40	APC1	165	160	0	97
				tank1	165	165	5	100

**Table 9.** Training data for Sequence L1618.

**Target (No. of Frames)**	**Filter**	**Working Frame Range**	**Training Frames**		**Filter Size**	***β***	***γ***	**Ψ**

			**Range Taken**	**Interval Taken**				
APC1 (291)	MACH Range-1	1–100	1–100	5	118 × 118	-	1.0	-
	MACH Range-2	101–200	101–200	5	118 × 118	-	1.0	-
	MACH Range-3	201–300	201–290	5	118 × 118	-	1.0	-
	EMACH Range-1	1–100	1–100	5	118 × 118	0.1	1.0	-
	EMACH Range-2	101–200	101–200	5	118 × 118	0.1	1.0	-
	EMACH Range-3	201–300	201–290	5	118 × 118	0.1	1.0	-

M60 (101)	MACH Range-1	1–300	1–100	5	118 × 118	-	1.0	-
	EMACH Range-1	1–300	1–100	5	118 × 118	0.1	1.0	-

Truck (6)	MACH Range-1	1–300	1–6	1	118 × 118	-	1.0	-
	EMACH Range-1	1–388	1–6	1	118 × 118	0.1	1.0	

-	DCCF Range-1	1–100	1–100	5	6 × 12	-	-	-
-	DCCF Range-2	101–200	201–300	5	8 × 16	-	-	-
-	DCCF Range-3	201–300	201–290	5	8 × 16	-	-	-
-	PDCCF Range-1	1–100	1–100	5	6 × 12	-	-	1.0, 1.5, 2.0
-	PDCCF Range-2	101–200	101–200	5	8 × 16	-	-	1.0, 1.5, 2.0
-	PDCCF Range-3	201–300	201–290	5	8 × 16	-	-	1.0, 1.5, 2.0

**Table 10.** Tracking results of three-target sequences.

**Seq. Name**	**Total Frames**	**No. of ROIs Taken**	**Threshold**	**Target Name**	**No. of Frames Target Present**	**No. of Frames Detected Correctly**	**Total No. of False Alarms**	**Percentage of Successful Detection**
MACH-DCCF Algorithm								

L1618	300	6	0.30, 0.52, 0.52	APC1	300	273	54	91

				M60	103	71	0	69

				truck	17	0	0	0

MACH-PDCCF Algorithm								

L1618	300	6	0.52	APC1	300	297	20	99

				M60	103	99	0	96

				truck	17	12	0	71

EMACH-PDCCF Algorithm								

L1618	300	4	0.52	APC1	300	297	23	99

				M60	103	99	0	96

				truck	17	12	0	71
